# William Frederick Bailey Eadon

**Published:** 1912-09

**Authors:** 


### Obituary.

### William Frederick Bailey Eadon, L.R.C.P., L.R.C.S. Edin.

Dr. Eadon, when he first came to Hambrook, was assistant to Dr. Day, later he became partner, and for the last thirty years has carried on the practice by himself.

His practice was a very extensive one, both as regards the number of patients and the distances travelled. At first he used horses to get about, but later he did all his work on an ordinary bicycle, often covering over fifty miles a day. For the last seven or eight years he had used a heavy-weight motor bicycle, far too heavy for a man of his physique. He rode this machine in all weathers, getting wet and covered with mud. One very wet day he called upon a patient of his, and they had a new servant who did not know the doctor. As they were expecting coal, the maid when he came to the front door told him to please take it round the back. He quite enjoyed the joke, and related it when he got to the bedroom of the patient. His practice was a very varied one. He had a large number of club patients, and also attended many of the best people in the district.

He never took a holiday. A patient once asked him why. He replied : " I do not feel I want one, and I am quite happy in my practice." He had no hobby, his whole heart was in his practice. He was an indefatigable worker, and never seemed happy unless he was busy.

He was held in very high esteem professionally, and people travelled long distances to get his opinion on their individual cases. Not only was he thought a great deal of in his professional capacity, but I have often heard people say they loved the little doctor. He was a good all-round general practitioner, and good to the poor people of the neighbourhood. He will be very much missed in this district.

Many years ago he was thrown from his trap ; except for this he was never ill enough to prevent him from doing his work.

In November, 1911, whilst in his surgery, he was seized with a fit, and was temporarily unconscious. He thought the attack was epileptic, but most probably it was due to slight cerebral hemorrhage. He stayed indoors a few days, but against advice he insisted on going out at the end of a week on his motor bicycle. Except for a few attacks of temporary hemianopsia, he continued fairly well until February of this year, and although it was pointed out to him how dangerous it was for him to ride, he would continue to do so.

In February, after having a lot of trouble with his motor bicycle, involving a good deal of stooping, he mounted his ordinary bicycle after a heavy morning surgery, and hurried to make up for lost time. He had another seizure whilst riding the bicycle, fell, cut his head, and was unconscious for some hours. When he recovered consciousness he had absolute homonymous hemianopsia in the right field of vision. After many weeks he got well enough (as he thought) to go out in his motor car and see a few patients ; but he only did so for a few days before having another attack. After this he had several attacks, and became hemiplegic. The last few days of his life he developed congestion of the bases of both lungs, which accelerated his death.

He never murmured during his illness, and was very patient, but I think he realised his life's work was done. I am sure he would rather have died when unable to do his own work. He said "he would rather wear out than rust out."

He died on August 22nd in his 64th year, and was buried at Downend Church on August 25th, when a large concourse of people attended the funeral.

